# Neuroprotective Effects of Estrogen Through BDNF-Transient Receptor Potential Channels 6 Signaling Pathway in the Hippocampus in a Rat Model of Perimenopausal Depression

**DOI:** 10.3389/fnagi.2022.869274

**Published:** 2022-07-08

**Authors:** Qiaoli Song, Weiming Huang, Wenbin Ye, Huan Yan, Liting Wang, Yan Yang, Xi Cheng, Weiqiang Zhang, Jie Zheng, Ping He, Yaojuan He, Dajun Fang, Xinjia Han

**Affiliations:** Department of Obstetrics and Gynecology, Guangzhou Women and Children’s Medical Center, Guangzhou Medical University, Guangzhou, China

**Keywords:** perimenopausal depression, estradiol, BDNF, TRPC6, neuronal excitability

## Abstract

Estradiol (E_2_) has been proven to be effective in treating perimenopausal depression (PD); however, the downstream signaling pathways have not been fully elucidated. Transient receptor potential channels 6 (TRPC6) plays a vital role in promoting neuronal development and the formation of excitatory synapses. At present, we found that the serum levels of E_2_ and brain-derived neurotrophic factor (BDNF) declined significantly in the women with PD compared to perimenopausal women, which was accompanied by a clear reduction in TRPC6 levels. To further reveal the effects of TRPC6 on neuronal survival and excitability, the PD-like rat model was established by the total removal of left ovary and 80% removal of right ovary followed by 21 days of the chronic unpredictable mild stress. Intragastric administration of E_2_ (2 mg/kg), intraperitoneal injection of BDNF/TrB signaling pathway inhibitor (K252a, 100 μg/kg) and TRPC6 agonist (OAG, 0.6 mg/kg), and intracerebroventricular infusion of anti-BDNF antibody for blocking BDNF (0.5 μg/24 μl/rat) daily for 21 days were conducted. The levels of BDNF and TRPC6 in rat serum were lower in PD rats compared to the control rats; the depression-like behavior was induced, the neuronal death rate in the hippocampus increased, and the thickness of postsynaptic density (PSD) and the number of asymmetric synapses decreased significantly in the PD group. E_2_ treatment greatly upregulated the serum levels of BDNF and TRPC6, the neuronal excitability indicated by an elevation in the PSD thickness and the numbers of asymmetric synapses, and these actions were reversed by K252a; co-administration of TRPC6 agonist and K252a improved neuronal degeneration and increased the neuronal excitability induced in the E_2_-treated PD rats. K252a or anti-BDNF antibody inhibited the increased neuronal BDNF and TRPC6 expression in E_2_-treated PD rats; co-treatment of TRPC6 agonist and anti-BDNF antibody reduced neuronal death and increased the BDNF and TRPC6 expression in the hippocampal CA1 neurons in the E_2_-treated PD rats. These results suggest that the neuroprotective role of E_2_ in PD is closely related to enhance the activity of BDNF/TRPC6 pathway and is helpful to provide new prevention and strategies.

## Introduction

Depression is the most important type of modern psychological disease and refers to a chronic and reoccurring neuropsychiatric disease. Epidemiological data have shown that the depression affects 350 million people each year all over the world currently (Uchida et al., 2018); especially, women are 2.5 times more prone to major depression than men (Weissman et al., 1984; Parker and Brotchie, 2010). Women who are aged 56 to 66 (the perimenopausal period) usually experience perimenopausal depression (PD); PD is a mood disorder and its main symptoms includes anxiety, stress, sleep disorders, tiredness, decline in concentration, and memory, accompanied by endocrine dysfunction and deterioration in ovarian function (Maki et al., 2018; Di et al., 2019; Willi and Ehlert, 2020). Mounting evidence has reveal that downregulated serum levels of estrogen contribute to the pathogenesis of PD (Callegari et al., 2007; Szpunar and Parry, 2018; Li D. et al., 2019; Slavich and Sacher, 2019). Several clinical trials showed that estrogen treatment dramatically improved depression-related behavior in perimenopausal women with depressive disorders than that in placebo controls (Schmidt et al., 2000; Soares et al., 2001).

The findings from clinical trials and animal model of PD indicate that serum levels of Estradiol (E_2_) fluctuating during the menopausal transition and psychosocial factors are a major cause of PD (Bethea and Reddy, 2015; Cao et al., 2019). Wendy et al. found that longer duration of estrogen exposure was significantly associated with a decreased risk of depression in women during the transition to menopause and postmenopause (Marsh et al., 2017). The protective actions of estrogen in PD are mainly through upregulating brain-derived neurotrophic factor (BDNF) levels. Serum levels of E_2_ and BDNF were lower in the women with depression in comparison with controls (Hui et al., 2016). In patients with perimenopausal syndrome, the serum BDNF level was much lower in depression group than that in non-depression group, and such level was negatively correlated with self-rating depression scale scores in the PD group (Guo et al., 2018). In the PD animal model which was established through ovariectomy followed chronic unpredictable mild stress (CUMS), both orcinol glucoside (Li et al., 2021) and optimized integration of fluoxetine and 7, 8-dihydroxyflavone (Amin et al., 2020) efficiently ameliorated depressive-like behaviors by upregulating the BDNF protein expression in the hippocampus. However, the mechanism of actions underlying the therapeutic effect of antidepressants of estrogen/BDNF is currently unclear.

Canonical transient receptor potential channels 6 (TRPC6) are non-selective, Ca^2+^-permeable cation channels in the central nervous system that play a critical role in growth cone guidance (State and Levitt, 2011), neuronal survival, synaptic plasticity (Li et al., 2005; Du et al., 2010; Lin et al., 2013b; Ko and Kang, 2017), spine morphology changes, and dendritic outgrowth (Leuner et al., 2007; Tai et al., 2008; Zhou et al., 2008). The dysregulation of TRPC6 has been reported to be involved in the pathophysiology of several neurodegenerative diseases such as Alzheimer’s disease, cerebral ischemia, epilepsy, depression, and so on. Significantly reduced TRPC6 mRNA levels in the blood cells were found in patients with Alzheimer’s disease compared to that age-matched controls (Lu et al., 2018). TRPC6 expression was dramatically downregulated in mouse cortices after ischemia that in sham mice (Wang et al., 2010). An obvious decline of TRPC6 was observed in the depression-like rats than in the control rats (Li et al., 2005). Studies from Zeng et al. (2015) demonstrated that TRPC6 protein levels were significantly higher in excised human epileptic cortex and hippocampus of mice with status epilepticus in comparison with those in controls. However, there has been far less focus on the changing pattern of TRPC6 and their roles in hippocampal plasticity in PD and whether the protective effects of estrogen treatment in PD are related to regulating TRPC6. This study would investigate the above two aspects from clinical samples and a rat model of PD.

## Materials and Methods

### Human Subjects and Clinical Serum Preparation

A total of 10 women diagnosed with PD and 10 age-matched control women were enrolled from Guangzhou Women and Children’s Medical Center. The recruitment date was from January 2019to March 2021. Kupperman Scale (a score of above 15), Self-Rating Anxiety Scale (SAS, a score of above 60), and Self-Rating Depression Scale (SDS, a score of above 60) were served as assessment criteria of depression diagnosis in code F32 according to the International Statistical Classification of Diseases and Related Health Problems (Marsh et al., 2017; Shimizu et al., 2019), and perimenopausal was defined as menstrual cycle length variability (change in menstrual cycle length of longer than 7 days, ≥60 days amenorrhea, or ≥2 skipped cycles in the last 12 months; No authors listed, 1996; Burger et al., 1999; Harlow et al., 2012). The average ages in the PD group and the control group were 48.8 ± 1.3 and 49.7 ± 1.2 years, respectively, in Table 1. Data on age, marital status, employment status, and complication showed no significant difference between the PD group and the control group in Table 1. Values on Kupperman, SAS, and SDS in the PD group showed that women had moderate depression and obviously higher than that in the control group (*p* < 0.001). On the second to third day of menstrual cycle or the endometrium was <5 mm thick as staged by a transvaginal Doppler ultrasound scan in all women, 3 ml fasting blood sample was obtained in sterile plain tubes without anticoagulant at 9:00–11:00 a.m. and centrifuged at 3,500 rpm at 4°C for 10 min to get serum; the serum was stored at –20°C until analysis. All participating women provided a written informed consent. The Ethics Committee of Guangzhou Women and Children’s Medical Center, Guangzhou Medical University, approved this study.

**TABLE 1:** Clinical characters of the study populations.

Characteristic	Depression (*n* = 10)	Control (*n* = 10)	*p*-value
Kupperman^a^	33.2 ± 3.6	9 ± 1.5	*p* < 0.001
SAS^a^	66.1 ± 1.9	39.5 ± 2.2	*p* < 0.001
SDS^a^	65.9 ± 2.8	37.9 ± 2.3	*p* < 0.001
Age, years^a^	48.8 ± 1.3	49.7 ± 1.2	NS
Marital status^b^			
Unmarried	10%	0%	*p* = 1.000
Married	90%	100%	
Employment status^b^			
Unemployed	40%	0%	*p* = 0.087
Employed	60%	100%	
Complication^b^			
Yes	20%	20%	NS
No	80%	80%	

*^a^The results were presented as mean ± SEM and analyzed by Mann–Whitney Test. NS: no significance.*

*^b^The results were presented as percentage and analyzed by Fisher’s Exact Test. NS: no significance.*

### Animals

A total of 80 female Sprague-Dawley rats weighing 250–270 *g* (2 months old) were obtained from Guangdong Medical Laboratory Animal Center, Guangzhou, China. The rats were housed in standard environmental conditions controlled at 22 ± 2°C, and 45–55% relative humidity, with 12:12-h light/dark cycle. Animals were provided with *ad libitum* access to food and water and allowed to 7-day habituation before experiments. All animal experiments were approved by the Animal Experiments Ethics Committee of Guangzhou Medical University and carried out in accordance with the institutional guidelines of the Animal Care and Use Committee of Guangzhou Medical University. Every effort was made to minimize the number of animals used and their suffering.

### Perimenopausal Depression-Like Rat Model and Drug Treatment

Animals were anesthetized by intraperitoneal injection of 10% chloral hydrate (320 mg/kg) and fixed in the prone position (Luo et al., 2015; Shi et al., 2021; Wei et al., 2021). In the lower third abdominal area, a 0.5–1 cm incision was made, and the entire left ovary and 80% of the right ovary were removed from the model rats; in the sham group, only the incision was performed; then, the muscle and skin layers were sutured separately. Each rat was placed in a single cage to allow for 3-day recovery.

The CUMS procedure was executed within 21 days; a total of 8 stimulations were as follows: wet sawdust bedding (24 h), cold water swimming (4°C), heat environment (45°C for 5 min), reversed light/dark cycle, food deprivation (24 h), water deprivation (24 h), and tail pinch (Zeng et al., 2015; Cao et al., 2019). Each stimulation was randomly selected per day and applied at least two times, and the same type of stimulation was performed discontinuously. The shame rats were separately housed.

The animals were randomly divided into eight groups (*n* = 9), control group (sham-operated rats treated with saline); OAG group (sham-operated rats treated with OAG); PD group (PD rats treated with saline); PD + E_2_ group (PD rats treated with estrogen); PD + E_2_ + K group (PD rats treated with estrogen) and K252a which serves as BDNF/TrkB signaling pathway antagonist; PD + E_2_ + K + O group (PD rats treated with estrogen, K252a, and TRPC6 agonist); PD + E_2_ + anti-BDNF group (PD rats treated with estrogen and anti-BDNF antibody); and PD + E_2_ + anti-BDNF + OAG group (PD rats treated with estrogen, anti-BDNF antibody, and TRPC6 agonist). All treatments were at the beginning of the stimulation conducted for 21 consecutive days. For the PD + E_2_ group, the PD rats received intragastric administration of estrogen (2 mg/kg) one time daily; following the above administration, in the PD + E_2_ + K group, the rats were intraperitoneally injected with BDNF/TrB signaling pathway antagonist (K252a, 100 μg/kg) one time daily; following the treatment in the PD + E_2_ + K group, rats in the PD + E_2_ + K + O group received intraperitoneal injection of TRPC6 agonist (OAG, 0.6 mg/kg) one time daily. The PD rats and sham-operated rats received intragastric administration and intraperitoneal injection of 0.9% saline one time at every 1-day interval.

For specifically blocking the BDNF signaling pathway, chicken anti-BDNF antibody (Cat#ARG65673, Arigo Biolaboratories, Shanghai, China) was intracerebroventricularly infused (Kleinridders et al., 2009; Jiang et al., 2017). Rats ere anesthetized with intraperitoneal injection of 10% chloral hydrate (0.3 ml/100 g) and fixed in a stereotaxic apparatus. The cannula was cemented in the left lateral brain ventricle (−0.2 mm anterior and 1.0 mm lateral relative to the bregma and 2.3 mm under the surface of the skull), and the incision was sutured. Animals were given 3 days to recover. Goat anti-BDNF antibody diluted in sterilized ACSF (final volume, 0.5 μg/24 μl/rat) daily for 21 days was delivered 0.3 μl/min by osmotic minipumps. Each brain infusion cannula was used to be attached an osmotic minipump.

### Sucrose Preference Test

Then, 4 days before CUMS procedure, each animal was given 1% sugar water instead of pure water for 2-day adaptation; 2 days before CUMS procedure, sucrose consumption procedure was performed; each animal was subjected to water and food deprivation for 23 h and provided with pure water for another 1 h. Then, one bottle of pure water and one bottle of 1% sugar water were put on the left side and the right side of each cage. Every 12 h, the bottles were switched. The sucrose consumption procedure was repeated on the last 2 days of each week during the 21-day stimulation period. The sucrose solution consumption percentage = 100% × sucrose solution intake/the total water consumption (Su et al., 2016; Zhu et al., 2019).

### Open Field Test

The black square box which was 500 mm × 500 mm enclosed by a wall of 400 mm in height was used for recording the animals. Each rat was placed in the middle of the box to freely explore their surroundings for 3 min. Tests were performed at days 1, 7, 14, and 21 of the experiment and the box was cleaned after each test. The total track length and duration time spent in the corner in the videos were analyzed using the opening activity experimental system of XR-XZ301 software (Shanghai Xinruan Information Technology Company, China).

### Tissue and Serum Preparation

At the end of the treatment, all rats were anesthetized by intraperitoneal injection of 10% chloral hydrate (0.3 ml/100 *g*), and blood samples were collected from the abdominal aorta in non-heparinized tubes and centrifuged at 3,500 rpm, 4°C for 10 min to obtain serum; the serum was stored at −20°C until analysis. Subsequently, the brain tissue was removed and one hemisphere was fixed in 4% paraformaldehyde for 3 days to make 4-μm paraffin sections from the coronal level (*n* = 6 for each group). The other hemisphere was immediately frozen in liquid nitrogen and stored at −80°C (*n* = 6 for each group). Brain tissues for electron microscopy were fixed in 2% glutaraldehyde and 2% paraformaldehyde in 0.15M phosphate buffer, pH 7.4 at 4°C for 1–2 day (*n* = 3 for each group).

### Detection of E_2_, Luteotropic Hormone, Follicle-Stimulating Hormone, BDNF, and Transient Receptor Potential Channels 6 Concentrations

Electrochemiluminescence assay kits and ELISA kits were used to determine the serum levels of E_2_, luteotropic hormone (LH), follicle-stimulating hormone (FSH), BDNF, and TRPC6 in patients with PD and age-matched controls and all experimental animals.

### Nissl Staining

Nissl staining was used to detect the degenerative degree of neurons. Briefly, 4-μm coronal sections were heated at 65°C for 45 min and dewaxed and rehydrated as follows: xylene I, xylene II, 100% alcohol, 95% alcohol, 85% alcohol, 75% alcohol, and 50% alcohol, 7 min for each procedure; sections were rinsed in distilled water for 2 × 7 min. Then, sections were immersed in Nissl staining solution (Cat#C0117, Beyotime Biotechnology, Shanghai, China) for 5 min and rinsed in distilled water for 2 × 1 min and dehydrated in 95% alcohol for 2 × 2 min, and clarified in xylene I and xylene II for 5 min. Sections were finally mounted with permanent mounting medium.

A total of 30 sections were analyzed for each group (6 animals per group, 5 sections per animal). A fluorescent microscope (Leica, DM4 B, Wetzlar, Germany) was used to capture the images of tissue sections from the hippocampus which were taken under the same magnification. ImageJ software (NIH, Bethesda, MD, United States) was applied to perform the quantitative analysis of neuronal numbers. The ratio of degenerative neurons was determined as the number of degenerative neurons divided by the total number of neurons.

### Fluoro-Jade B Staining

Fluoro-Jade B staining (Cat# TR-150,Thebarton, South Australia) was performed to identify degenerated neurons (Schmued and Hopkins, 2000). The starting procedure was the same as the dewaxing and hydration methods for Nissl staining. Then, the brain sections were incubated in a solution of 1% NaOH diluted in 80% ethanol for 6 min and then put in 70% ethanol for 2 min and distilled water for 2 min. Sections were then incubated in a solution with 0.06% potassium permanganate for 15 min, washed with distilled water for 2 min, and stained with 0.0004% FJB solution in 0.1% acetic acid for 20 min. Sections were washed with distilled water 3 times and dried at 50°C. Then, sections were cleared in xylene for 5 min and mounted with DPX (Cat#PN4330, G-CLONE Biotechnology Company, Beijing, China). A total of 35 sections for each group (7 animals per group, 5 sections per animal) were analyzed. Micrographs were taken from the hippocampus under the same magnification using a fluorescent microscope (Leica, DM4 B, Wetzlar, Germany). The number of degenerated neurons was quantitatively analyzed by ImageJ software (NIH, Bethesda, MD, United States).

### Double Immunofluorescent Staining

After sections were processed with dewaxing, hydration, and antigen repair treatment, they were rinsed in distilled water and 0.01M PBS separately for 5 min. Blocking solution with 1% BAS and 0.3% Triton in 0.01M PBS was added in sections for 2 h at room temperature, and then, sections were incubated in primary antibodies diluted by blocking solution at 4°C for 30 h. Sections were rinsed in 0.01M PBS for 3 times (5 min each) and incubated in secondary antibodies diluted by blocking solution at room temperature for 3 h. Then, sections were washed with 0.01M PBS again and stained with DAPI solution and mounted by anti-fluorescence quenching agent. The information for primary antibodies was as follows: the neuronal marker was NeuN (mouse, cat#66836, 1:30, Proteintech Group, Rosemont, IL, United States), BDNF (rabbit, 1:50, cat#bs-4989R, Bioss Biotechnology), and TRPC6 (rabbit, 1:50, cat#bs-21380R, Bioss Biotechnology, Beijing, China). The information for secondary antibodies was as follows: goat anti-rabbit (1:500, cat# ab150081, Alexa Fluor 488) and goat anti-mouse (1:500, cat# ab150116, Alexa Fluor 594).

The JACoP plugin of ImageJ software was applied to analyze all intracellular puncta within each immunofluorescent micrograph (Dunn et al., 2011; Wang et al., 2017), and 20 sections from each group were used (4 sections/sample, *n* = 5). The results were expressed relative to the data from the control group.

### Electron Microscopy

Hippocampal tissues were fixed in 2.5% glutaraldehyde and 2% paraformaldehyde in 0.15M PB for 1–2 days at 4°C. Then, tissues were post-fixed in cold 0.5% osmium tetroxide for 2 h and dehydrated in 30% alcohol, 50% alcohol, 70% alcohol, and 95% for 10 min each and in 100% alcohol for 3 × 10 min. Then, tissues were rinsed in propylene oxide for 3 × 20 min and embedded in EMbed 812 (Electron Microscope Sciences) at 60°C for 48 h to be made into resin blocks. Semi-thin sections (1–2 μm) were cut from each block on an ultramicrotome and stained with 1% toluidine blue to determine the observed areas under light microscope. Then, 50–70-nm-ultrathin sections collected on 200 mesh grids were stained in 3% uranyl acetate for 30 min and 2.66% lead citrate for 15 min. Micrographs were captured using a Philips 400 transmission electron microscope.

According to the methods in our previous studies, (Ruan et al., 2012; Han et al., 2016), to better understand the morphological changes in neurons and excitatory synapses in the hippocampus from each experimental rats, 3 ultrathin sections from 3 animals per group (3 blocks) were prepared. The total field in each section was taken from left to right to achieve micrographs. A total of 15–20 images from each group were used to quantify the number of excitatory synapses. The active zone containing docked synaptic vesicles facing the postsynaptic membrane was recognized as a presynaptic terminal, and the postsynaptic density (PSD) area was determined from the postsynaptic membrane to the interface toward the cytoplasm. ImageJ software was used to measure the changes in number of excitatory synapses and the thickness of PSD in the hippocampus in all experimental group.

### Statistical Analysis

Data were presented as mean ± SEM. One-way analysis of variance was used to compare the differences among multiple groups and was followed by an appropriate least significant difference *post hoc* test (if homogeneity test of variance was neat) or Dunnett’s test (if homogeneity test of variance was not neat). The SPSS 17.0 software package (SPSS, Chicago, IL, United States) was used to perform the statistical analyses. A *p*-value of 0.05 was considered statistically significant.

## Results

### The Serum Levels of E_2_, Luteotropic Hormone, Follicle-Stimulating Hormone, BDNF, and Transient Receptor Potential Channels 6 Between the Control and Depression Group in Perimenopausal Women

**FIGURE 1:**
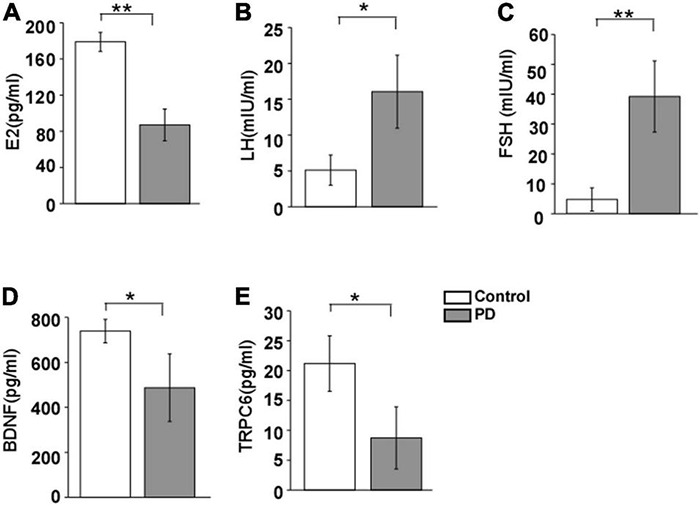
Changes in the E_2_ **(A)**, LH **(B)**, FSH **(C)**, BDNF **(D)**, and TRPC6 **(E)** levels in serum in PD women and age-matched control women (*n* = 20, each group). E_2_, estradiol; FSH, follicle-stimulating hormone; and LH, Luteotropic hormone. Results are presented as mean ± SEM. *p < 0.05; **p < 0.01.

The levels of E_2_ and BDNF in serum were significantly lower in the PD women than that in the control women (E_2_: 87.1 ± 17.6 pg/ml vs. 179.3 ± 10.5 pg/ml, *F* = 20.32, *p* = 0.002; BDNF: 486.2 ± 150.1 pg/ml vs. 738.9 ± 51.8 pg/ml, *F* = 10.88, *p* = 0.035, Figure 1). The LH and FSH levels demonstrated an obvious rising trend in the PD group compared to the control group (LH: 16.1 ± 5.1 mIU/ml vs. 5.3 ± 2.1 mIU/ml, *F* = 7.05, *p* = 0.046; FSH: 39.2 ± 11.9 mIU/ml vs. 4.7 ± 3.9 mIU/ml, *F* = 24.41, *p* = 0.009, Figure 1). The TRPC6 levels were clearly reduced in the PD group compared to the control group (8.7 ± 5.2 pg/ml vs. 21.2 ± 4.6 pg/ml, *F* = 8.29, *p* = 0.043), and such changing trend was the same as that of BDNF and E_2_.

### Effects of E_2_, BDNF/TrkB Signaling Pathway AntAgonist (K252a), and Transient Receptor Potential Channels 6 Agonist on Serum Biochemical Indicators in the Perimenopausal Depression Rats

**FIGURE 2:**
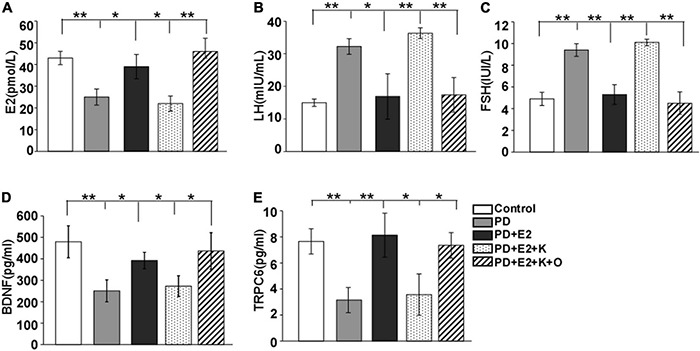
Changes in the E_2_ **(A)**, LH **(B)**, FSH **(C)**, BDNF **(D)**, and TRPC6 **(E)** levels in serum in all experimental groups (*n* = 7, each group). Results are presented as mean ± SEM. *p < 0.05; **p < 0.01.

Compared with the control rats (as shown in Figure 2), the serum levels of E_2_, BDNF, and TRPC6 decreased greatly in the PD rats (E_2_: 25.4 ± 3.7 pmol/L vs. 43.3 ± 3.1 pmol/L, *F* = 14.1, *p* = 0.006; BDNF: 250.8 ± 51.1 pg/ml vs. 479.4 ± 74.3 pg/ml, *F* = 37.4, *p* = 0.008; and TRPC6: 3.15 ± 0.97 pg/ml vs. 7.65 ± 0.98 pg/ml, *F* = 14.6, *p* = 0.007). However, the levels of LH and FSH in serum were significantly induced in the PD rats, which kept consistence with hormonal changes in PD women (LH: 32.2 ± 2.38 mIU/ml vs. 14.9 ± 1.1 mIU/ml, *F* = 43.03, *p* = 0.001; FSH: 9.4 ± 0.58 IU/L vs. 5.0 ± 0.61 IU/L, *F* = 28.53, *p* = 0.001). In the PD + E_2_ group, E_2_ treatment significantly increased the BDNF (392.1 ± 38.3 pg/ml, *F* = 6.12, *p* = 0.049) and TRPC6 (8.1 ± 1.7 pg/ml, *F* = 12.33, *p* = 0.0097) concentrations, but decreased the LH (16.8 ± 6.9 mIU/ml, *F* = 6.31, *p* = 0.04) and FSH (5.3 ± 0.9 IU/L, *F* = 14.37, *p* = 0.009) concentrations than that in the PD group. In the PD + E_2_ + K group, K252a could remarkably reduce the E_2_, BDNF, and TRPC6 levels (22.7 ± 3.5 pmol/L, *F* = 6.6, *p* = 0.033; 272.6 ± 48.3 pg/ml, *F* = 6.55, *p* = 0.046; 3.6 ± 1.6 pg/ml, *F* = 5.18, *p* = 0.043) in comparison with the PD + E_2_ group. In the PD + E_2_ + K + O group, TRPC6 (TRPC6 agonist) could clearly upregulate the E_2_, BDNF, and TRPC6 levels (46.3 ± 6.1 pmol/L, *F* = 11.58, *p* = 0.009; 436.8 ± 85.1 pg/ml, *F* = 6.88, *p* = 0.04; 7.4 ± 0.9 pg/ml, *F* = 5.43, *p* = 0.041) than that in the PD + E_2_ + K group. Whereas the LH and FSH levels in the PD + E_2_ + K group showed a similar level to that in the PD group, such levels clearly decreased in PD + E_2_ + K + O group (LH: 17.3 ± 5.3 mIU/ml vs. 36.4 ± 1.6 mIU/ml, *F* = 20.9, *p* = 0.005; FSH: 4.5 ± 1.0 IU/L vs.10.1 ± 0.3 IU/L, *F* = 18.3, *p* = 0.007).

### Increasing Transient Receptor Potential Channels 6 Activity Could Reverse the Effects on Behavioral Changes in BDNF/TrkB Signaling Pathway Antagonist (K252a) on E_2_ Treatment in the Rat Model of Perimenopausal Depression

**FIGURE 3:**
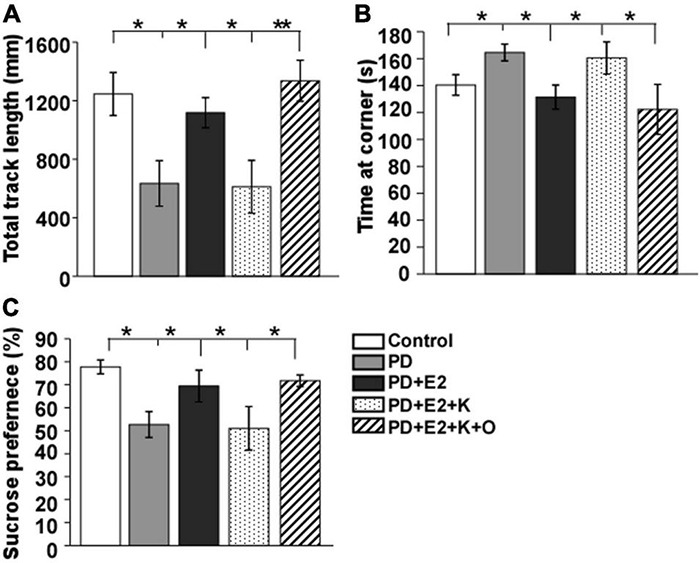
Histograms showing statistical analysis of the total track length **(A)** and time at the corner in the open field test **(B)** and the percentage of sucrose solution Consumption **(C)**. Results are presented as mean ± SEM. (*n* = 8, each group). *p < 0.05; **p < 0.01.

The depression-like behavior in animals was tested using open-field test and sucrose solution consumption procedure (as shown in Figure 3). The total movement distance decreased and the time at the corner increased obviously in the PD rats in comparison with that in the control group (634.7 ± 155.7 vs. 1245.9 ± 146.5 mm, *F* = 8.21, *p* = 0.021; 164.5 ± 6.3 vs. 140.4 ± 7.6 s, *F* = 5.94, *p* = 0.041); the sucrose solution consumption percentage was clearly reduced in the PD rats (52.6% ± 5.6 vs. 77.7% ± 3.0, *F* = 14.7, *p* = 0.04). These values in the PD group were the similar to that in the E_2_ and K252a-treated group (611.5 ± 180.8 mm; 160.5 ± 11.9 s; 51.0% ± 9.5), which showed a significant difference compared to those in the PD + E_2_ group and PD + E_2_ + K + O groups (1,117.7 ± 103.1 mm, *F* = 5.90, *p* = 0.041 and 1,336.3 ± 139.9 mm, *F* = 18.7, *p* = 0.007; 131.4 ± 8.9 s, *F* = 8.73, *p* = 0.039 and 122.3 ± 18.6 s, *F* = 7.3, *p* = 0.046; 69.4% ± 6.9, *F* = 10.5, *p* = 0.049 and 71.7% ± 2.5, *F* = 17.4, *p* = 0.034).

### Transient Receptor Potential Channels 6 Agonist Attenuated Neuronal Death in the Hippocampal CA1 Area Induced by Inhibiting BDNF Activity in the E_2_-Treated Perimenopausal Depression Rats

**FIGURE 4:**
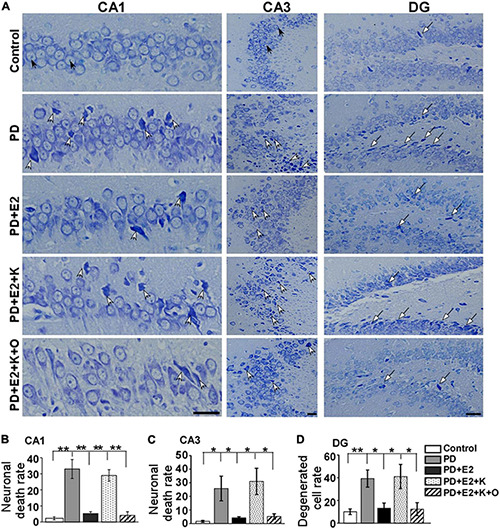
Nissl staining showed morphological changes of neurons in the hippocampus from all experimental groups. **(A)** White arrows indicated destroyed neurons, black arrows indicated normal neurons and long arrows indicated degenerated dentate gyrus (DG) cells. Histograms showed the changes in neuronal death rate and degenerated cell rate in the hippocampal CA1 **(B)**, CA3 **(C)**, and DG **(D)** areas in all animal groups (*n* = 6, each group). Results are presented as mean ± SEM. *p < 0.05; **p < 0.01. Scale bar = 30 μm.

Normal neurons which had large cell bodies, were rich in cytoplasm, and had one or two large round nuclei were observed in the control group (Figure 4A). In other groups, shrunken cell bodies and dark cytoplasm were clearly seen in the degenerative neurons. The damaged neurons seemed much more in the PD and PD + E_2_ + K group than that in the control, PD + E_2_, and PD + E_2_ + K + O groups. Quantitative analysis showed the same trend among all groups (Figure 4B). In the control group, the neuronal death rate in the CA1 region was 2.3% ± 0.9, and this rate increased to 33.6% ± 5.9 in the PD group (*F* = 25.3, *p* = 0.02). In the PD + E_2_ group, such rate (5.3% ± 1.2) was lower than that in the PD group (*F* = 20.4, *p* = 0.003). Additionally, after K252a administration, this rate increased to 29.3% ± 3.6 (*F* = 38.3, *p* = 0.000). In the PD + E_2_ + K + O group, this rate (4.1% ± 2.3) was similar to that in the PD + E_2_ group and decreased significantly than in the PD + E_2_ + K group (*F* = 33, *p* = 0.000, Figure 4B). The neuronal death rate in the CA3 area is much higher in the PD group than that in the control group (25.8% ± 9.1 vs. 1.6% ± 0.7, *F* = 7.03, *p* = 0.03, Figure 4C); E_2_ administration could significantly decrease such rate (4.14% ± 0.89) compared to the PD group (*F* = 5.6, *p* = 0.045). In the PD + E_2_ + K group, such rate (31% ± 9.7) was higher than that in the PD + E_2_ group (*F* = 7.6, *p* = 0.025); further TRPC6 agonist treatment significantly decreased such rate (5.2% ± 2.1, *F* = 6.8, *p* = 0.031). The degenerated dentate gyrus cell rate increased remarkably in the PD group compared to the control group (39.2% ± 7.5 vs. 10.1% ± 2.5, *F* = 13.46, *p* = 0.006, Figure 4D); E_2_ administration could significantly decrease such rate (13.1% ± 4.6) compared to the PD group (*F* = 8.7, *p* = 0.018); after K252a administration, this rate increased to 41.1% ± 10.7 (*F* = 5.74, *p* = 0.043); further TRPC6 agonist treatment significantly reduced such rate (12.3% ± 5.7, *F* = 5.6, *p* = 0.045).

### Activating Transient Receptor Potential Channels 6 Could Increase the Thickness of Postsynaptic Densitys in the Hippocampus Induced by BDNF/TrkB Signaling Pathway Antagonist (K252a) on E_2_-Treated Perimenopausal Depression Rats

**FIGURE 5:**
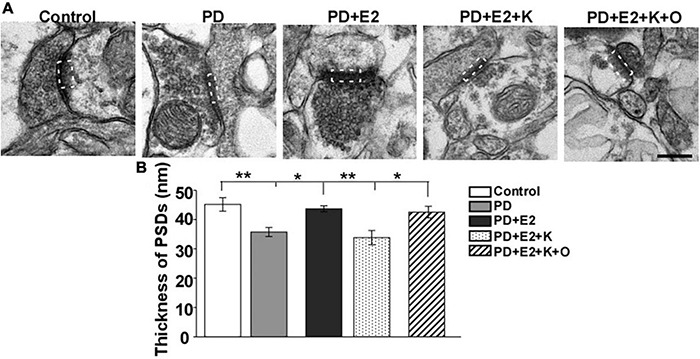
**(A)** Electron micrographs and histograms **(B)** showing the changes in the thickness of PSD in the hippocampus in all experimental groups. The dotted box indicated the postsynaptic dense area (*n* = 3, each group). Results are presented as mean ± SEM. *p < 0.05; **p < 0.01. Scale bar = 250 nm.

The thickness of PSD on single photographs was randomly analyzed, and changes in PSD thickness may reflect the changes in neuronal excitability (Ruan et al., 2012). Therefore, we further investigated the PSD thickness in the hippocampus by electron microscope technology. In the PD group, the PSD became thinner than that in the control group, and E_2_ treatment increased the PSD thickness; following K252a treatment, the thickness of PSD seemed similar to that in the PD group; TRPC6 agonist could reverse the actions of K252a in the E_2_ treated-PD rats (Figure 5A). Quantitative analysis demonstrated that the PSD thickness in the PD group (35.7 ± 4.6 nm) was lower than that in the P group (45.1 ± 2.3 nm, *F* = 11.87, *p* = 0.009, Figure 5B). Such value in the PD + E_2_ (43.6 ± 1.0 nm) group increased greatly compared with that in the PE group (*F* = 4.37, *p* = 0.021). The PSD thickness in the PD + E_2_ (*F* = 14.3, *p* = 0.005) and PD + E_2_ + K + O (42.5 ± 1.9 nm, *F* = 7.5, *p* = 0.025) groups was higher than that in the PD + E_2_ + K group (33.8 ± 2.4 nm, Figure 5B).

### Transient Receptor Potential Channels 6 Agonist Increased the Reduced Number of Asymmetric Synapses in the Hippocampal Area Induced by Inhibiting BDNF Activity in the E_2_-Treated Perimenopausal Depression Rats

**FIGURE 6:**
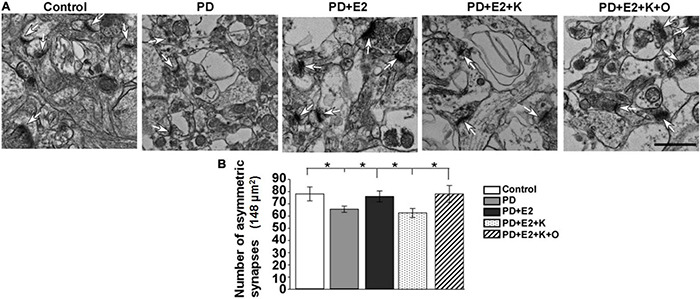
**(A)** Electron micrographs and histograms **(B)** showing the changes in the number of asymmetric synapses in the hippocampus in all experimental groups. White arrowheads indicated the typical asymmetric synapses (*n* = 3, each group). Results are presented as mean ± SEM. *p < 0.05. Scale bar = 1e p.

More than 90% of glutamatergic fibers form asymmetric synapses on dendrites and somas of pyramidal neurons in the hippocampus (Takacs et al., 2012); increase in the number of asymmetric synapses suggested the enhancement of excitatory synaptic transmission in the hippocampus. The changing pattern of asymmetric synapses in all experimental groups in this study is consistent with that of PSD thickness. In the PD group, the number of asymmetric synapses became less than that in the control group, and E_2_ treatment increased such number; following BDNF/TrkB signaling pathway antagonist (K252a) treatment, the number of asymmetric synapses decreased to a level similar to that in the PD group; TRPC6 agonist could reverse the actions of K252a in the E_2_ treated-PD rats (Figure 6A). In an area (148 μm^2^)in each micrograph, a total number of synapses of the control group were higher in the *P* group (77.6 ± 5.7) than in the PD group (65.1 ± 2.6; *F* = 5.75, *p* = 0.043). Such number in the PD + E_2_ group (75.6 ± 4.5) was significantly higher than those in the PD (*F* = 5.55, *p* = 0.047). The number of asymmetric synapses was lower in the PD + E_2_ + K group (62.5 ± 3.7) than that in the PD + E_2_ (*F* = 5.7, *p* = 0.045) and PD + E_2_ + K + O (78.1 ± 6.9 nm, *F* = 6.3, *p* = 0.04, Figure 6B).

### Ultrastructural Analysis of Neurons in all Experimental Groups

**FIGURE 7:**
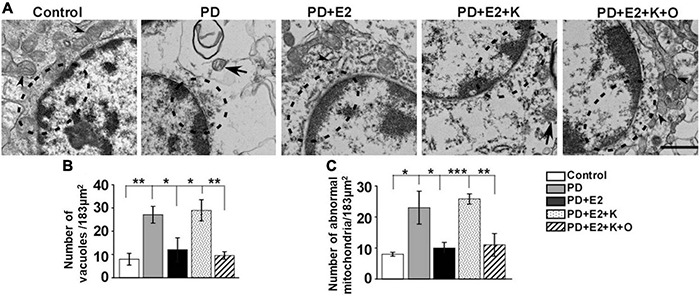
Transmission electron microscopy of hippocampus in the different groups. **(A)** Neurons in the control group were normal, while neurons in the PD group seemed degenerated with discontinuous nucleus membrane; a large number of vacuoles appeared in the cytoplasm; the destroyed neurons were also seen in the PD + E_2_ + K group. The morphology of neurons in the control group was similar to that of the PD + E_2_ and PD + E_2_ + K + O groups. The dotted boxes indicated the morphological changes of the nuclear membrane; arrowheads indicated normal mitochondria; arrows with lines indicated abnormal mitochondria. Scale bar = 1 μm. Histograms showing the changes in number of vacuoles **(B)** and abnormal mitochondria **(C)** in the control group, PD group, PD + E_2_ group, PD + E_2_ + K group, and PD + E_2_ + K + O group (*n* = 3, each group). Results are presented as mean ± SEM. *p < 0.05; **p < 0.01; ***p < 0.001.

Our electron microscopy results showed that hippocampal neurons in the control group seemed normal, mitochondrial cristae was clearly observed in the cytoplasm, and complete nucleus membrane and chromatin with clear nucleoli were clearly visible (Figure 7A). However, in the PD group, the nucleus membrane and perinuclear organelles morphology seemed to be destroyed, mitochondrial cristae became unclear, and abnormal mitochondria was observed. E_2_ treatment obviously improved the degeneration of the nuclear membrane and decreased the damaged degree of mitochondria in the PD group; inhibiting BDNF activity could reverse the effects of E_2_ on morphological changes in neurons; in the PD + E_2_ + K + O group, TRPC6 agonist could further decrease the degeneration of neurons, the nuclear membrane seemed intact, normally structural mitochondria around the nuclear membrane was obviously seen, and less cavity in the cytoplasm was observed in the PD + E_2_ + K + O and PD + E_2_ groups than that in the PD + E_2_ + K group (Figure 7A). The number of vacuoles and abnormal mitochondria in each photograph (an area of 183 μm^2^) was counted in Figures 7B,C the quantitative method was based on our previous studies and studies from other research groups (Ruan et al., 2012; Kang et al., 2019; Konan et al., 2019). In the control group, the number of vacuoles was 7.9 ± 2.5, and this number increased to 27.1 ± 3.6 in the PD group (*F* = 18.86, *p* = 0.002); E_2_ treatment in the PD group greatly inhibited such number to 12.1 ± 5.1 (*F* = 5.75, *p* = 0.043); further K252a treatment reversed such number to 28.9 ± 4.5 (*F* = 6.08, *p* = 0.039); in the PD + E_2_ + K + O group, this number decreased to 9.5 ± 1.6 (*F* = 16.08, *p* = 0.004, Figure 7B). The number of abnormal mitochondria in the PD group was obviously higher than that in the control group (23.1 ± 5.3 vs. 8.0 ± 0.63, *F* = 7.89, *p* = 0.023, Figure 7C); such number was lower in the PD + E_2_ group (10.0 ± 1.81, *F* = 5.38, *p* = 0.049) than that in the PD group; this number was higher in the PD + E_2_ + K group (25.8 ± 1.65, *F* = 41.3, *p* = 0.000) than that in the PD + E_2_ group. The number of abnormal mitochondria in the PD + E_2_ + K + O group decreased to 11.0 ± 3.67 (*F* = 13.48, *p* = 0.006) compared to that in the PD + E_2_ + K group (Figure 7C).

### BDNF/TrkB Inhibitor (K252a) and Anti-bDNF Antibody Notably Antagonized the Inhibition of Neuronal Death in the Hippocampus in the Perimenopausal Depression-Like Rats Treated by E_2_

**FIGURE 8:**
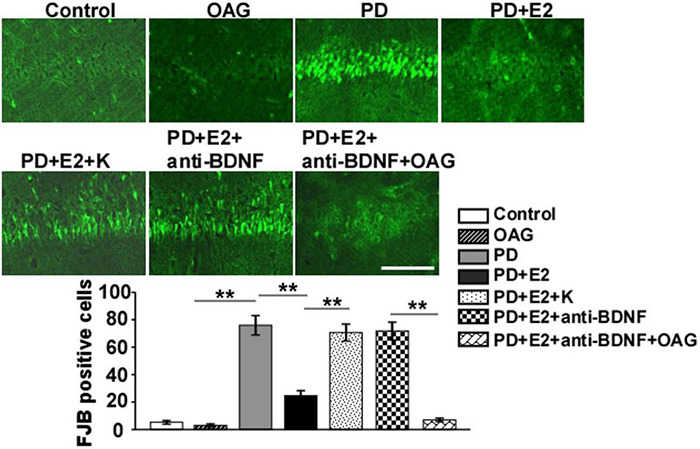
Representative micrographs showing the changes in neuronal death by FJB staining of the hippocampal CA1 area in the control group, OAG group, PD group, PD + E_2_ group, PD + E_2_ + K group, PD + E_2_ + anti-BDNF group, and PD + E_2_ + anti-BDNF + OAG group. Quantitative analysis of the FJB-positive cells in the CA1 (*n* = 7). All data are expressed as means ± SEM. (*n* = 7, each group). **p < 0.01. Scale bar = 100 μm.

To assess the effects of treatment with K252a or anti-BDNF antibody or co-treatment of anti-BDNF antibody and TRPC6 agonist (OAG) on the hippocampal neuronal death in the CA1 area following E_2_ administration in the PD-like model, FJB staining was conducted (Figure 8). In the PD group, the number of FJB-labeled cells (76 ± 7.1) was higher than that in the control (5.2 ± 1.3, *F* = 93.97, *p* = 0.000) and OAG (3.0 ± 1.1, *F* = 101.1, *p* = 0.000) groups. Such value was markedly reduced in the PD + E_2_ group (24.6 ± 3.7, *F* = 40.4, *p* = 0.000) in comparison with the PD group; co-treatment with K252a (70.6 ± 6.2, *F* = 39.9, *p* = 0.000) and anti-BDNF antibody (71.8 ± 6.5, *F* = 39.2, *p* = 0.000) both greatly increased neuronal death. The number of neuronal death was significantly decreased in the PD + E_2_ + anti-BDNF + OAG (7.0 ± 1.3, *F* = 94.0, *p* = 0.000, Figure 8) group than that in the PD + E_2_ + anti-BDNF group.

### BDNF/TrkB Inhibitor (K252a) and Anti-bDNF Antibody Notably Antagonized the Increase in BDNF and Transient Receptor Potential Channels 6 Expression in the Hippocampal CA1 Neurons in the Perimenopausal Depression-Like Rats Treated by E_2_

**FIGURE 9:**
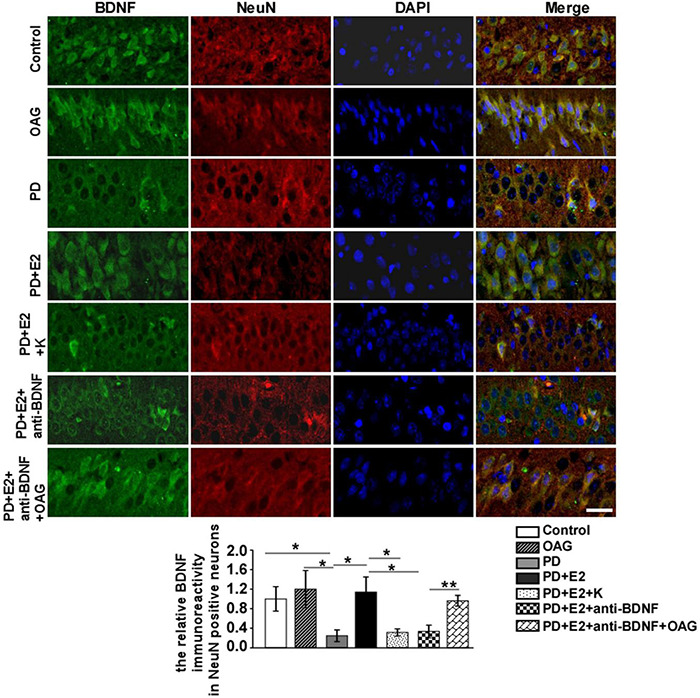
K252a or anti-BDNF administration significantly inhibited the upregulation of BDNF expression in the hippocampal CA1 area in PD rats treated by E_2_; further co-treatment of TRPC6 agonist blocked the above effects. Immunofluorescent micrographs showing the changes in fluorescence intensity of BDNF in NeuN-labeled neurons in the CA1 region of the hippocampus in the control group, OAG group, PD group, PD + E_2_ group, PD + E_2_ + K group, PD + E_2_ + anti-BDNF group, and PD + E_2_ + anti-BDNF + OAG group. The relative immunoreactivity of BDNF in neurons was quantified (*n* = 5, each group). Data are presented as means + SEM. *p < 0.05; **p < 0.01. Scale bar = 30 μm.

**FIGURE 10:**
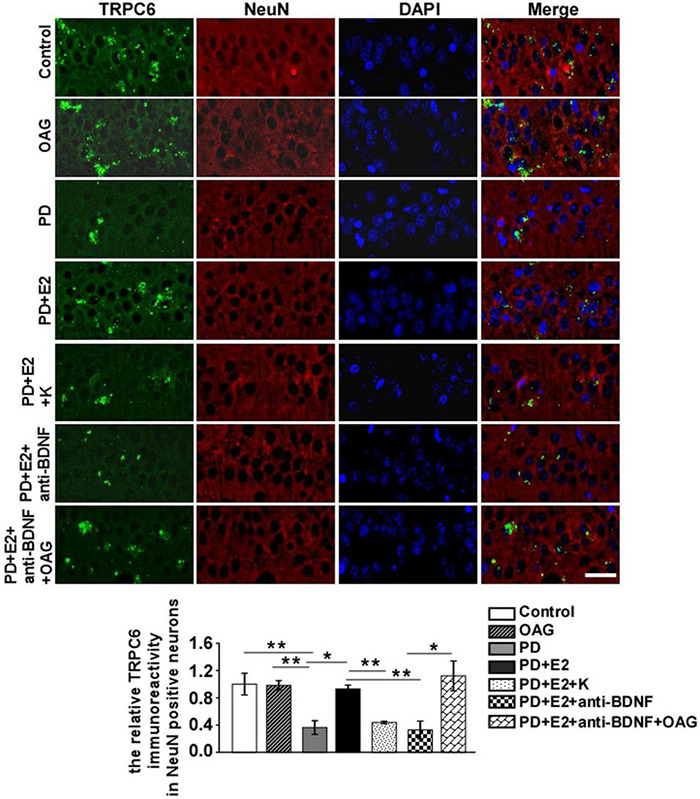
K252a or anti-BDNF administration significantly inhibited the upregulation of TRPC6 expression in the hippocampal CA1 area in PD rats treated by E_2_; further co-treatment of TRPC6 agonist blocked the above effects. Immunofluorescent micrographs showing the changes in fluorescence intensity of TRPC6 in NeuN-labeled neurons in the CA1 region of the hippocampus in the control group, OAG group, PD group, PD + E_2_ group, PD + E_2_ + K group, PD + E_2_ + anti-BDNF group, and PD + E_2_ + anti-BDNF + OAG group. The relative immunoreactivity of TRPC6 in neurons was quantified (*n* = 5, each group). Data are presented as means + SEM. *p < 0.05; **p < 0.01. Scale bar = 30 μm.

To detect the effects of treatment with K252a or anti-BDNF antibody or co-treatment of anti-BDNF antibody and TRPC6 agonist (OAG) on the hippocampal BDNF and TRPC6 expression in the CA1 area following E_2_ administration in the PD-like model, double immunofluorescent staining was performed (Figures 9, 10). In the PD group, the BDNF (0.25 ± 0.12) and TRPC6 (0.36 ± 0.10) immunoreactivities were lower than that in the control (BDNF, 1.0 ± 0.25, *F* = 7.61, *p* = 0.025; TRPC6, 1.0 ± 0.16, *F* = 11.16, *p* = 0.001) and OAG (BDNF, 1.2 ± 0.38, *F* = 5.51, *p* = 0.047; TRPC6, 0.98 ± 0.07, *F* = 16.6, *p* = 0.004) groups. Such value was obviously increased in the PD + E_2_ group (BDNF, 1.14 ± 0.31, *F* = 6.99, *p* = 0.029; TRPC6, 0.93 ± 0.057, *F* = 5.43, *p* = 0.048) in comparison with the PD group; co-treatment with K252a (BDNF, 0.31 ± 0.073, *F* = 6.44, *p* = 0.035; TRPC6, 0.44 ± 0.019, *F* = 67.54, *p* = 0.000) and anti-BDNF antibody (BDNF, 0.33 ± 0.13, *F* = 5.51, *p* = 0.047; TRPC6, 0.328 ± 0.13, *F* = 16.6, *p* = 0.004) both greatly reduced the neuronal BDNF and TRPC6 expression. The neuronal BDNF and TRPC6 expression was significantly enhanced in the PD + E_2_ + anti-BDNF + OAG (BDNF, 0.96 ± 0.11, *F* = 12.6, *p* = 0.007, Figure 9; TRPC6, 1.12 ± 0.22, *F* = 9.33, *p* = 0.016, Figure 10) group than that in the PD + E_2_ + anti-BDNF group.

## Discussion

Studies have shown that TRPC3 and TRPC6 channels are novel and essential mediators through which BDNF promotes neuronal survival and dendritic remodeling. *In vitro* experiments found that BDNF prevented cerebellar granule neurons against apoptosis induced by serum deprivation; such protection was suppressed through blocking TRPC3 or TRPC6 (Jia et al., 2007). BDNF application in cultured hippocampal neurons enhanced TRPC3 expression; the increase in dendritic spine density in CA1 pyramidal neurons of hippocampal slice stimulated by BDNF was prevented by TRPC3 inhibitor (Amaral and Pozzo-Miller, 2007). BDNF-triggered axonal growth cones turning in cultured cerebellar granule cells could be abolished by downregulating TRPC6 expression *via* short interfering RNA (Amaral and Pozzo-Miller, 2007). In this study, we found that serum levels of E_2_, TRPC6, and BDNF decreased dramatically in patients with PD when compared to those that in normal controls; in the rat PD model, E_2_ treatment increased the downregulated expression of TRPC6 and BDNF in hippocampal neurons; BDNF/TrkB signaling pathway inhibitor (K252a) could reverse the effects of estrogen; furthermore, TRPC6 agonist could upregulated the reduced expressions of TRPC6 and BDNF caused by E_2_ in combination with K252a. The number of degenerative neurons increased greatly in the PD rats in comparison with that in the control rats; E_2_ treatment reduced the neuronal degeneration, which could be abolished by K252a; further administration of TRPC6 agonist alleviated neuronal injury in the hippocampus. Our data suggest that estrogen treatment can protect hippocampal neurons from injury in the rat PD model and the underlying mechanisms are partly through increasing BDNF/TRPC6 pathway.

Overexpression of TRPC6 in bone marrow stromal cells *via* a CRISPR-based synergistic activation mediator could decrease brain injury significantly and improve neurofunctional outcomes in a rat model of ischemia/reperfusion (Li W. et al., 2019). Increasing TRPC6 activity by the TRPC6 agonist (hyperforin) dramatically reduced Aβ levels which were a major pathological factor for Alzheimer’s disease and ameliorated cognitive performance (Lu et al., 2018). The TRPC6 agonist (hyperforin) could recover the decreased TRPC6 protein expression in the hippocampus in a rat depression model and reduced the number of denatured neurons; moreover, the impaired spatial cognitive ability detected by Morris water maze was rescued and rats’ sucrose solution intake was higher in the depression group treated by hyperforin (Liu Y. et al., 2015). Immunostaining results demonstrated that TRPC6 expression was markedly increased in the cortex and hippocampus, and spatial learning and memory in Morris water maze were enhanced in TRPC6 transgenic mice (Zhou et al., 2008).

In a rat depression model, impaired cognitive ability along with decreased hippocampal TRPC6 expression was observed (Liu Y. et al., 2015). In the mice depression model induced by corticosterone, a long-lasting antidepressant-like activity was induced by hyperforin by directly activating TRPC6 (Pochwat et al., 2018). These findings indicated that decreased TRPC6 expression closely contributed to the pathogenesis of depression disorder. This study found that the survival neurons in the hippocampus decreased significantly in the PD-like rats than that in sham rats; estrogen treatment could recover the decreased TRPC6 expression and the declined number of survival neurons and alleviate the increased anxiety in new environment in the PD group. These actions of estrogen were reversed by K252a, and further application of TRPC6 agonist could counteract the effects of K252a in some extent. These results reveal that enhancement of TRPC6 expression in the hippocampus can improve PD-like behavior; another mechanism of the neuroprotective roles of estrogen-BDNF pathway in PD is closely related to decreased neuronal damage due to re-upregulated TRPC6 levels.

Immunofluorescent staining with the indicated antibodies showed that TRPC6 was partially colocalized with PSD-95 in the postsynaptic sites of hippocampal neurons and TRPC6 had an important role in synaptic development, learning, and memory (Zhou et al., 2008). Mouse primary hippocampal neurons transduced by shRNA targeting TRPC6 showed less spine density and fewer synapses (Griesi-Oliveira et al., 2015). Hyperforin (which can invoke TRPC6 activation) markedly increased the proportion of mature stubby spines and decreased the proportion of immature thin spines in CA1 pyramidal neurons compared to vehicle controls from hippocampal slice cultures by activating TRPC6 channels (Leuner et al., 2013). In the hippocampus of mice with pilocarpine-induced status epilepticus, a significant increase in protein expressions of TRPC3 and TRPC6 was found in comparison with those in controls (Leuner et al., 2013); using anti-TRPC6 antibody to inhibit TRPC6 expression in status epilepticus, mice cause synaptic reorganization characterized by reduced dendritic arborization and spine density of hippocampal CA3 area (Leuner et al., 2013). In the depression-like rats, Golgi-staining showed shorter dendritic length of hippocampal neuron and DiI staining showed lower neurite spine density than that in the control group and the hyperforin-treated stressed group (Liu Y. et al., 2015). Turning of growth cones in cultured cerebellar granule cells induced by BDNF can be inhibited by knockdown TRPC6 (Amaral and Pozzo-Miller, 2007).

Several studies have investigated the downstream signaling pathway of TRPC6, and Jian et al. found that TRPC6 could promote the formation of excitatory synapses through activating CaMKIV-CREB pathway (Zhou et al., 2008). In the cortical area of patients with focal cortical dysplasia, both the mRNA and protein levels of TRPC6 and BDNF were increased compared to that of normal control cortex; higher expression of calmodulin-dependent kinase IV (CaMKIV) which is the downstream molecule of TRPC6 was observed in the lesion area (Zheng et al., 2016); most TRPC6-positive cells were glutamatergic neurons which were excitatory. TRPC6 knockdown resulted in mitochondrial elongation in the DGC degeneration by decreasing extracellular signal-regulated kinase 1/2 (ERK1/2) phosphorylation (Ko and Kang, 2017). Resveratrol significantly attenuated infarct volumes and enhanced neurological scores at 24 h after ischemia/reperfusion injury, and such effects were closely related to increase the activity of the TRPC6-MEK-CREB and TRPC6-CaMKIV-CREB pathways (Lin et al., 2013a). In this study, the neuroprotective actions of estrogen in PD are partly through increasing the expressions of BDNF and TRPC6; though the above results indicated the downstream molecular mechanism of activating TRPC6 in the hippocampal neurons of PD rats, we need to further investigate the specific targets of upregulating TRPC6 activity through *in vitro* intervention experiments.

The PD-like rat model used in this study was the typical perimenopausal model (Tian et al., 2019) in combination with CUMS model (Guan et al., 2021), and such PD model was commonly used in exploring the effects and mechanism actions of some drugs such as curculigoside and Icariin in treating PD (Miao et al., 2017; Cao et al., 2019). The decrease in E_2_ level and an increase in FSH level together with longer residence time at the corner by open field test caused by the removals of rat ovaries and stimulation by CUMS in this study were the key symptoms for PD (Salazar-Pousada et al., 2017). Many other perimenopausal animal models were applied: hot flash animal models of menopausal women (Federici et al., 2016); the loss of ovarian small follicles and intact ovaries caused by the occupational chemical 4-vinylcyclohexene diepoxide could induce a perimenopause stage in rats (Brooks et al., 2016) and so on. In this study, complete deletion of left ovary and 80% deletion of right ovary were performed to induce perimenopausal model, which led to a sharp decrease in E_2_. Ovaries of perimenopausal women were intact and E_2_ levels were gradually decreasing; 20% of right ovary left in PD rats can mimic the gradual decline in E_2_. In this study, from the perspective of neuroscience and gynecology, we investigated the neuroprotective effects of estrogen through BDNF-TRPC6 signaling pathway in the hippocampus in a rat model of PD, and the typical perimenopausal model (Tian et al., 2019) in combination with CUMS (Guan et al., 2021) is stable and suitable for our research purpose.

BDNF plays a major role in the pathophysiology of depression and the treatment of antidepressants mainly by binding to its TrkB receptor (Duman and Li, 2012). The alleviative depressive symptoms in the forced swim test, sucrose preference test, upregulation of BDNF/TrkB signaling, and hippocampal neurogenesis by BDNF treatment in CUMS were completely abolished by TrkB antagonist K252a (Wang et al., 2014; Liu B. B. et al., 2015; Yan et al., 2016; Guan et al., 2021). To detect whether BDNF is necessary for axonal growth in cultured synapsins I knockout neurons, K252a was also used and reversed the increase in the length and number of axonal branching induced by BDNF (Marte et al., 2017). To specifically block the BDNF/TrkB signaling pathway, anti-BDNF neutralizing antibody was intracerebroventricularly infused in chronic social defeat stress (CSDS) model (Jiang et al., 2015, 2017) and CUMS model (Guan et al., 2021), and such antibody was subcutaneously administered using osmotic pumps placed on the back in spinal cord injury (Wada et al., 2019). It was worth mentioning that both the anti-BDNF antibody and the TrkB inhibitor (K252a) were applied to explore whether the antidepressant effects of some drugs were through the activation of BDNF/TrkB signaling pathway (Jiang et al., 2015; Guan et al., 2021); intraperitoneal injection of K252a improved the sucrose preference index approximately by 46% and intracerebroventricular infusion of anti-BDNF antibody increased the sucrose preference index nearly by 40% in CUMS model (Guan et al., 2021); both intraperitoneal injection of K252a and intracerebroventricular infusion of anti-BDNF antibody improved the sucrose preference index approximately by 28% in CSDS model (Jiang et al., 2015); in addition, the increased protein expression of BDNF in the hippocampus by drugs was reduced nearly by 34% in CUMS model (Guan et al., 2021) and 40% in CSDS model following intraperitoneal injection of K252a (Jiang et al., 2015). Intracerebroventricular infusion of K252a decreased the neurogenesis in the hippocampus of adult rats induced by intermittent hypoxia approximately by 45% and intracerebroventricular infusion of anti-BDNF antibody decreased such neurogenesis approximately by 42% (Zhu et al., 2010). The above findings indicated that intraperitoneal or intracerebroventricular injection of K252a could mimic the blocking effects of anti-BDNF antibody on BDNF signaling pathway in some extent. In this study, the increased neuronal death and a decline in BDNF and TRPC6 expression in the hippocampus found in PD rats were improved by E_2_ treatment, and these changes were greatly abolished by K252a and anti-BDNF antibody infusion; further application of TRPC6 agonist could reverse the effects of anti-BDNF antibody on regulating BDNF and TRPC6 expression; in addition, further application of TRPC6 agonist could reverse the effects of both K252a and anti-BDNF antibody on neurodegeneration. Our results suggested that BDNF-TRPC6 signaling pathway was closely involved in the anti-depression effects of E_2_ in PD model. However, more *in vivo* and *in vitro* experiments related to the depletion or compensation of BDNF and TRPC6 signaling in PD are needed to solidify our present results.

## Data Availability Statement

The original contributions presented in this study are included in the article/supplementary material; further inquiries can be directed to the corresponding authors.

## Ethics Statement

The studies involving human participants were reviewed and approved by the Ethics Committee of the Guangzhou Women and Children’s Medical Center approved the study (Nos. 2018041701 and 201922200). The patients/participants provided their written informed consent to participate in this study. The animal study was reviewed and approved by all procedures were approved by the Guangzhou Medical University Animal Ethics Committee (Permit Number: 2012–50). Written informed consent was obtained from the individual(s) for the publication of any potentially identifiable images or data included in this article.

## Author Contributions

QS, WH, WY, HY, LW, YY, XC, WZ, PH, YH, DF, and XH collected the samples. QS, WH, WY, and XH conducted the experiments. XH, QS, WH, PH, YH, DF, and HY analyzed the data. XH, QS, PH, YH, and DF conceived and designed the experiments. XH and WH wrote the manuscript. All authors contributed to the article and approved the submitted version.

## Conflict of Interest

The authors declare that the research was conducted in the absence of any commercial or financial relationships that could be construed as a potential conflict of interest.

## Publisher’s Note

All claims expressed in this article are solely those of the authors and do not necessarily represent those of their affiliated organizations, or those of the publisher, the editors and the reviewers. Any product that may be evaluated in this article, or claim that may be made by its manufacturer, is not guaranteed or endorsed by the publisher.
